# Supplementary material for: Evaluation of umbilical cord blood serum eye drops for severe dry eye in two distinct populations—Sjögren’s syndrome and mustard gas–induced ocular injury: Protocol for a pilot randomized clinical trial
Source: PLoS One. 2025 Dec 4;20(12):e0337186. doi: 10.1371/journal.pone.0337186 (PMC12677545; doi:10.1371/journal.pone.0337186)
Supplement: S3 File — (DOCX) [file pone.0337186.s003.docx]

**Title:
Evaluation of the Effect of Umbilical Cord Blood Serum Eye Drops in the Treatment of Severe Dry Eye Symptoms in Patients with Sjögren’s Syndrome and Mustard Gas Chemical Veterans**

**Principal Investigator / Supervisor:
Seyed Hashem Daryabari**

**Research Project Executor:
Masoud Rostami**

**Affiliation:
School of Medicine, Chemical Injuries Research Center, Baqiyatallah Hospital (Clinical Research Development Unit), Elite Center and Conscription Reduction Program**

**Proposal Abstract**

1. **Project Title:
   Evaluation of the Effect of Umbilical Cord Blood Serum Eye Drops in the Treatment of Severe Dry Eye Symptoms in Patients with Sjögren’s Syndrome and Mustard Gas Chemical Veterans**
2. **Principal Investigator / Project Manager:
   Seyed Hashem Daryabari**
3. **Program Code:
   F2R3P2S9**
4. **Alignment with Roadmap / Organizational Mission:
   Yes**
5. **Expected Output / Deliverable:
   Yes (Scientific Article)**

**Problem Statement**

**(Referencing existing studies and documentation, describe the problem, its scope and current status, the importance and necessity of addressing it, a brief history of the issue, contributing or related factors, and its health, economic, social, etc. impacts.)**

Sjögren's syndrome is a chronic autoimmune disease that initially affects mucosal exocrine glands, most notably the salivary and lacrimal glands. Consequently, patients typically experience dry mouth and dry eyes, although the disease is often accompanied by musculoskeletal disorders and damage to other organ systems (1). Diagnosis of Sjögren’s syndrome is based on a combination of sicca symptoms and the presence of autoimmune features, such as T cell activity (confirmed by positive salivary gland biopsy) or B cell activity (identified by autoantibodies) (2). In most cases, Sjögren’s syndrome occurs without other inflammatory or autoimmune diseases, referred to as primary Sjögren’s syndrome. However, it can also occur alongside other autoimmune conditions, including thyroid disease, rheumatoid vasculitis, and systemic lupus erythematosus, referred to as secondary Sjögren’s syndrome (3).

There is an increased prevalence of dry eye among patients with autoimmune diseases, including Sjögren’s syndrome, affecting approximately 8% of the population, of whom 78% are women (4, 5). Dry eye is generally due to instability of the tear film, in which lipid layer thickness may be a determining factor (6), and is exacerbated by chronic inflammation, decreased aqueous secretion, and increased evaporation (7). Patients may complain of foreign body sensation, pain, photophobia, blurred vision, and difficulty opening their eyes (8).

Tears contain various growth factors and essential components, including epidermal growth factor, vitamin A, neurotrophic factors, albumin, and both specific and nonspecific antimicrobial agents such as lysozyme, lactoferrin, α-lysin, and other proteins that play a crucial role in maintaining the health of the ocular surface epithelium (9, 10). Lysozyme hydrolyzes peptidoglycan in bacterial cell walls, providing antibacterial action, and its ability to degrade chitin also confers antifungal properties (10, 11). α-lysin disrupts cell membranes through an unclear mechanism, and its concentration in tears exceeds that in serum and plasma (12). Lactoferrin reversibly binds two iron atoms, thereby limiting bacterial access to iron for growth and metabolism (13). Specific antimicrobial agents in tears include immunoglobulins A, G, and M (14).

The tear film provides a smooth refractive surface and nourishment for the cornea and plays a key role in ocular defense. Any decrease in tear production or alteration in tear composition leads to ocular surface damage and pathology (15).

Long-term ocular complications from chemical warfare agents include photophobia (73.2%), decreased vision (72.5%), dry eye sensation (66.4%), foreign body sensation (61.1%), epiphora (46.3%), and pain (43%). Slit lamp findings reveal meibomian gland dysfunction (96%), blepharitis, tear film abnormalities, and ocular surface dryness (80–90%). Chronic blepharitis and reduced tear secretion are major contributors to the progression of ocular complications from chemical agents. The risk of chemical keratopathy increases with higher initial exposure, greater disability percentage, and longer duration of ocular involvement (21).

Although the initial ocular effects of exposure to chemical warfare agents include tearing, edema, ocular discharge, and even blindness, these may gradually resolve within days or weeks, but residual keratopathy may develop, resulting in long-term effects (22). Such patients commonly suffer from dry eye complications.

Conventional treatments for dry eye syndrome include artificial tears, topical corticosteroids, cyclosporine A, therapeutic contact lenses, protective glasses, punctal occlusion, and tarsorrhaphy. However, these treatments do not supply the essential growth factors and components naturally present in tears (16).

Umbilical cord blood serum eye drops, derived from the blood of healthy mothers’ umbilical cords, are rich in cytokines, vitamin A, growth factors, neurotrophic factors, essential tear components, as well as antibacterial and antifungal agents such as IgG, lysozyme, and others. These drops create a suitable environment for cell and tissue repair and regeneration. They are preservative-free, safe, and effective in promoting recovery and regeneration of damaged cells and tissues, especially in healing and restoring the ocular epithelium (9, 17–19).

Human umbilical cord blood serum eye drops are safe, rich in growth factors, essential tear components, defensive and neurotrophic factors. In addition to providing vital tear components, they support the repair and regeneration of damaged tissues. They are structurally most similar to natural tears and have demonstrated promising effects in the treatment of dry eye and ocular surface disorders. However, the specific therapeutic effects of these drops in treating patients with Sjögren’s syndrome—characterized by lacrimal gland dysfunction, tear deficiency, and ocular surface involvement—and in chemical warfare veterans who experience severe dry eye and tissue injuries affecting the external eye (cornea, conjunctiva, eyelids), have not been comprehensively evaluated. These patients frequently display moderate to severe tissue damage due to chronic, severe dry eye.

Therefore, this study aims to evaluate the effects of umbilical cord blood serum eye drops on healing and regenerating damaged ocular surface tissues, improving vision impaired by ocular surface tissue destruction, and alleviating severe dry eye symptoms in patients with Sjögren’s syndrome and chemical warfare veterans. Both subjective and objective methods will be used for assessment.

Moreover, the results of this study could be applicable to other similar ocular surface conditions involving severe dry eye and tissue injury, such as Stevens-Johnson syndrome, keratoconjunctivitis sicca, burns, and damage from chemical or industrial agents.

**Literature Review**

A study conducted in 2006 by Yoon and colleagues titled “Evaluation of the Effect of Umbilical Cord Blood Serum Eye Drops in the Treatment of Severe Dry Eye” involved 31 patients with severe dry eye who were treated with umbilical cord blood serum eye drops. The study concluded that umbilical cord serum contains essential tear components and that the eye drops are safe and effective for treating severe dry eye (20).

In a 2007 study by Yoon et al., titled “Therapeutic Effect of Umbilical Cord Blood Serum Eye Drops on Dry Eye Associated with Graft-Versus-Host Disease (GVHD),” 24 eyes from 12 patients suffering from severe dry eye due to GVHD were treated with 20% umbilical cord blood serum eye drops. No significant side effects were observed following treatment. The study indicated that these eye drops are safe and may be an effective method for managing severe dry eye related to GVHD (21).

In 2014, Erdem and colleagues conducted a study entitled “Therapeutic Effect of Umbilical Cord Blood Serum Eye Drops in Patients with Persistent Epithelial Defects (PED),” involving 16 eyes from 14 patients resistant to conventional therapies. These patients were treated with 20% umbilical cord blood serum eye drops. The results showed that the drops are safe and effective in managing persistent epithelial defects, and the severity of the disease was a determining factor in the recovery time (22).

In 2022, Kumar and colleagues conducted a pilot study titled “Therapeutic Profile of Umbilical Cord Blood Serum and Patient-Derived Serum in the Treatment of Ocular Surface Disorders.” The study included 101 eyes with ocular surface disorders caused by dry eye disease, chemical burns, and ocular allergy. The results demonstrated that umbilical cord blood serum was more effective than autologous serum in promoting ocular surface reconstruction (23).

Additionally, in 2007, Yoon et al. conducted a study entitled “Use of Umbilical Cord Blood Serum Eye Drops in the Treatment of Neurotrophic Keratitis,” involving 28 patients with neurotrophic keratitis resistant to standard treatments. These patients were treated with 20% umbilical cord blood serum eye drops, 6 to 10 times daily. The study concluded that umbilical cord blood serum is rich in neurotrophic factors, and these eye drops represent an effective treatment for neurotrophic keratitis (24).

**Significance and Necessity of the Study / Novelty of the Proposal**

Based on previous studies, the use of umbilical cord blood serum eye drops appears to be a safe and effective method for treating the symptoms of dry eye and for promoting the repair and regeneration of ocular surface tissue damage. Recent research has increasingly focused on the beneficial tissue-regenerating effects of this approach and on the improvement of both subjective and objective symptoms of dry eye, particularly in severe cases associated with various underlying diseases.

Therefore, evaluating the efficacy of this therapeutic method in alleviating severe dry eye symptoms in patients with Sjögren’s syndrome and chemical warfare veterans—and utilizing the findings for the treatment of severe dry eye and tissue damage resulting from this syndrome and similar conditions in the country—is of great necessity.

This study’s innovative aspect lies in its focus on assessing the therapeutic effects of umbilical cord blood serum eye drops specifically in two challenging patient populations: those with Sjögren’s syndrome and those with severe ocular complications due to chemical exposure, where there is a significant need for effective, restorative treatments.

**Scientific References and Background**

The following sources have been cited to support the background, significance, and methodology of the present proposal:

1. Fox RI. Sjögren's syndrome. The Lancet. 2005;366(9482):321-31.
2. Aggarwal R, Anaya J-M, Koelsch KA, Kurien BT, Scofield RH. Association between secondary and primary Sjögren’s syndrome in a large collection of lupus families. Autoimmune Diseases. 2015;2015.
3. Amador-Patarroyo MJ, Arbelaez JG, Mantilla RD, Rodriguez-Rodriguez A, Cárdenas-Roldán J, Pineda-Tamayo R, et al. Sjögren’s syndrome at the crossroad of polyautoimmunity. Journal of autoimmunity. 2012;39(3):199-205.
4. Fox RI, Howell FV, Bone RC, Michelson PE, editors. Primary Sjogren syndrome: clinical and immunopathologic features. Seminars in arthritis and rheumatism; 1984: Elsevier.
5. Fairweather D, Frisancho-Kiss S, Rose NR. Sex differences in autoimmune disease from a pathological perspective. The American journal of pathology. 2008;173(3):600-9.
6. Isreb M, Greiner J, Korb D, Glonek T, Mody S, Finnemore V, et al. Correlation of lipid layer thickness measurements with fluorescein tear film break-up time and Schirmer's test. Eye. 2003;17(1):79-83.
7. Sotozono C, Ueta M, Yokoi N. Severe dry eye with combined mechanisms is involved in the ocular sequelae of SJS/TEN at the chronic stage. Investigative Ophthalmology & Visual Science. 2018;59(14):DES80-DES6.
8. Susiyanti M, Kurnia DA, Fasha I, Irawati Y, Rachmadi L, Liem IK, et al. Treatment of Severe Dry Eye in Stevens-Johnson Syndrome with Umbilical Cord Serum Eye Drops. Clinical Ophthalmology. 2022:4089-95.
9. Yoon K-C, Heo H, Im S-K, You I-C, Kim Y-H, Park Y-G. Comparison of autologous serum and umbilical cord serum eye drops for dry eye syndrome. American journal of ophthalmology. 2007;144(1):86-92. e2.
10. Bron A, Seal D. The defences of the ocular surface. Transactions of the Ophthalmological Societies of the United Kingdom. 1986;105:18-25.
11. Repaske R. Lysis of gram-negative bacteria by lysozyme. Biochimica et biophysica acta. 1956;22(1):189-91.
12. Ford LC, DeLange RJ, Petty RW. Identification of a nonlysozymal bactericidal factor (beta lysin) in human tears and aqueous humor. American journal of ophthalmology. 1976;81(1):30-3.
13. Selinger DS, Selinger RC, Reed WP. Resistance to infection of the external eye: the role of tears. Survey of ophthalmology. 1979;24(1):33-8.
14. German A, Hall E, Day M. Measurement of IgG, IgM and IgA concentrations in canine serum, saliva, tears and bile. Veterinary immunology and immunopathology. 1998;64(2):107-21.
15. Davidson HJ, Kuonen VJ. The tear film and ocular mucins. Veterinary ophthalmology. 2004;7(2):71-7.
16. Noble BA, Loh RS, MacLennan S, Pesudovs K, Reynolds A, Bridges L, et al. Comparison of autologous serum eye drops with conventional therapy in a randomised controlled crossover trial for ocular surface disease. British Journal of Ophthalmology. 2004;88(5):647-52.
17. Giannaccare G, Buzzi M, Fresina M, Velati C, Versura P. Efficacy of 2-month treatment with cord blood serum eye drops in ocular surface disease: an in vivo confocal microscopy study. Cornea. 2017;36(8):915-21.
18. Ang LP-K, Do TP, Thein ZM, Reza HM, Tan X-W, Yap C, et al. Ex Vivo Expansion of Conjunctival and Limbal Epithelial Cells Using Cord Blood Serum–Supplemented Culture Medium. Investigative ophthalmology & visual science. 2011;52(9):6138-47.
19. Yoon KC. Use of umbilical cord serum in ophthalmology. Chonnam medical journal. 2014;50(3):82-5.
20. Foulks GN, Forstot SL, Donshik PC, Forstot JZ, Goldstein MH, Lemp MA, et al. Clinical guidelines for management of dry eye associated with Sjögren disease. The ocular surface. 2015;13(2):118-32.
21. Ghasemi H, Ghazanfari T, Ghassemi-Broumand M, Javadi MA, Babaei M, Soroush MR, et al. Long-term ocular consequences of sulfur mustard in seriously eye-injured war veterans. Cutaneous and ocular toxicology. 2009;28(2):71-7.
22. Etezad‐Razavi M, Mahmoudi M, Hefazi M, Balali‐Mood M. Delayed ocular complications of mustard gas poisoning and the relationship with respiratory and cutaneous complications. Clinical & experimental ophthalmology. 2006;34(4):342-6.
23. Yoon K-C, Im S-K, Park Y-G, Jung Y-D, Yang S-Y, Choi J. Application of umbilical cord serum eyedrops for the treatment of dry eye syndrome. Cornea. 2006;25(3):268-72.
24. Yoon K, Jeong I, Im S, Park Y, Kim H, Choi J. Therapeutic effect of umbilical cord serum eyedrops for the treatment of dry eye associated with graft-versus-host disease. Bone marrow transplantation. 2007;39(4):231-5.
25. Erdem E, Yagmur M, Harbiyeli I, Taylan-Sekeroglu H, Ersoz R. Umbilical cord blood serum therapy for the management of persistent corneal epithelial defects. International journal of ophthalmology. 2014;7(5):807.
26. Kumar A, Chaurasiya D, Sultan S, Soni D, Kubrey S, Singh P, et al. Therapeutic Profile of Human Umbilical Cord Blood Serum and Autologous Serum Therapies in Treatment of Ocular Surface Disorders: A Pilot Study. Journal of Ocular Pharmacology and Therapeutics. 2023;39(1):36-47.
27. Yoon K-C, You I-C, Im S-K, Jeong T-S, Park Y-G, Choi J. Application of umbilical cord serum eyedrops for the treatment of neurotrophic keratitis. Ophthalmology. 2007;114(9):1637-42. e2.
28. Pakdel F, Gohari MR, Jazayeri AS, Amani A, Pirmarzdashti N, Aghaee H. Validation of farsi translation of the ocular surface disease index. Journal of ophthalmic & vision research. 2017;12(3):301.
29. Sharma N, Lathi SS, Sehra SV, Agarwal T, Sinha R, Titiyal JS, et al. Comparison of umbilical cord serum and amniotic membrane transplantation in acute ocular chemical burns. British Journal of Ophthalmology. 2015;99(5):669-73.
30. Kallarackal G, Ansari E, Amos N, Martin J, Lane C, Camilleri J. A comparative study to assess the clinical use of Fluorescein Meniscus Time (FMT) with Tear Break up Time (TBUT) and Schirmer’s tests (ST) in the diagnosis of dry eyes. Eye. 2002;16(5):594-600.
31. Whitcher JP, Shiboski CH, Shiboski SC, Heidenreich AM, Kitagawa K, Zhang S, et al. A simplified quantitative method for assessing keratoconjunctivitis sicca from the Sjögren's Syndrome International Registry. American journal of ophthalmology. 2010;149(3):405-15.

**Definitions of Terms**

**Sjögren’s Syndrome:**
A chronic autoimmune disease primarily characterized by dysfunction of the salivary and lacrimal glands, resulting in symptoms of dry mouth and dry eyes.

**Primary Sjögren’s Syndrome:**
In most cases, Sjögren’s syndrome occurs independently, without the presence of other inflammatory or autoimmune diseases.

**Secondary Sjögren’s Syndrome:**
Sjögren’s syndrome may also occur in association with other autoimmune diseases, such as thyroid disease, rheumatoid vasculitis, or systemic lupus erythematosus.

**Dry Eye:**
A condition generally caused by instability of the tear film and chronic inflammation, often in combination with reduced aqueous secretion and increased evaporation.

**Tear (Tear Fluid):**
A complex fluid containing various growth factors and essential components, including epidermal growth factor, vitamin A, neurotrophic factors, albumin, and both specific and nonspecific antimicrobial agents such as lysozyme, lactoferrin, α-lysin, and other protein compounds with nonspecific antimicrobial activity.

**Lysozyme:**
An enzyme that destroys bacteria by hydrolyzing the peptidoglycan in bacterial cell walls. Due to its ability to degrade chitin, lysozyme also possesses antifungal properties.

**Alpha-lysin (α-lysin):**
A protein that causes disruption of cell membranes via an unknown mechanism. Its concentration in tears is higher than in serum or plasma.

**Lactoferrin:**
A protein that reversibly binds two iron atoms, thereby restricting bacterial access to iron necessary for growth and metabolism.

**Umbilical Cord Blood Serum Eye Drops:**
Eye drops derived from umbilical cord blood, containing large amounts of cytokines, vitamin A, growth factors, neurotrophic factors, essential tear components, and antimicrobial and antifungal agents such as IgG, lysozyme, and other compounds. These preservative-free drops are considered safe and effective in treating severe dry eye and epithelial disorders.

**5. Specific Objectives**

1. To determine the effect of umbilical cord blood serum eye drops on improving visual acuity in patients with dry eye syndrome.
2. To determine the effect of umbilical cord blood serum eye drops on improving the symptoms of dry eye in patients with dry eye syndrome.
3. To determine the effect of umbilical cord blood serum eye drops on the quality of the tear film in patients with dry eye.
4. To determine the effect of umbilical cord blood serum eye drops on the rate of epithelial regeneration in patients with dry eye.

**General Objective**

To determine the effect of umbilical cord blood serum eye drops in treating the symptoms of dry eye.

**Practical Objectives**

Given the nature of severe dry eye disease and associated ocular surface disorders, and considering the limitations of conventional treatments and the high similarity of umbilical cord blood serum eye drops to natural tear components—as well as their richness in growth and neurotrophic factors—an accurate evaluation of these drops in improving dry eye symptoms and repairing damaged ocular surfaces is essential. This is particularly important given their safety, accessibility, and efficacy in the country. The findings from this study could enhance treatment protocols for severe dry eye, especially in patients with Sjögren’s syndrome and chemical warfare veterans, and may also be applicable to the management of dry eye and tissue damage caused by chemical warfare and industrial agents.

**Hypotheses / Research Questions**

1. Does the use of umbilical cord blood serum eye drops improve the symptoms of dry eye?
2. Does the use of umbilical cord blood serum improve visual acuity in patients with dry eye syndrome?
3. Does administration of umbilical cord blood serum eye drops enhance the quality of the tear film in patients with dry eye?
4. Is the use of umbilical cord blood serum eye drops effective in promoting epithelial regeneration in patients with dry eye?

**Expected Outcomes**

The outcomes of this study include the publication of the results in scientific articles and the application of the findings to improve treatment protocols for the management of severe dry eye resulting from diseases and conditions associated with severe dry eye and ocular surface tissue damage. These conditions include, but are not limited to, Sjögren’s syndrome, Stevens-Johnson syndrome, and chemical or industrial burns and injuries.

**Study Methods**

**Study Design:**
This study is a controlled clinical trial with a control group and two follow-up measurement time points.

**Study Population:**
The study population will consist of patients with Sjögren’s syndrome-related dry eye and individuals with ocular injuries due to mustard gas exposure, referred to the ophthalmology clinic at Baqiyatallah Hospital.

- **Study Location:**
  Ophthalmology Clinic, Baqiyatallah (AJ) Hospital
- **Inclusion Criteria:**
  Patients with Sjögren’s syndrome and severe dry eye, as well as individuals with chemical ocular injuries due to mustard gas exposure who do not respond well to standard dry eye treatments, will be included. Additional criteria are a tear break-up time (TBUT) of less than 5 seconds, a Schirmer test result less than 5 mm, and positive fluorescein staining (score ≥3).
- **Exclusion Criteria:**
  Patients with infections or inflammation unrelated to dry eye, contact lens users, those with ocular allergies, a history of ocular surgery, eyelid or eyelash abnormalities, as well as pregnant or breastfeeding women, will be excluded from the study.
- **Data Collection Instruments:**
  - **Slit Lamp (Streit-Haag BQ 900, USA):** For qualitative assessment of anterior and posterior eye segments (using a 90D lens for posterior segment evaluation)
  - **Auto-refractometer (KR-8100; Topcon, Tokyo, Japan):** For determination of refractive errors
  - **Retinoscope (Heine Beta 200, Optotechnic HEINE, Germany):** For determination of refractive errors
  - **LED Visual Acuity Chart (MEDISIZE, Korea SMART LC 13):** For assessment of visual acuity
  - **Trial Lens Set (Magnon TF-600 Inami lens, JAPAN):** For numerical determination of dioptric power
  - **Trial Frame (Inami Trial Frame – 0391, JAPAN):** For placement of trial lenses
  - **Required Supplies:** Fluorescein strips and Schirmer test paper strips
- **Validity of Instruments/Data Collection:**
  All tests will be conducted using instruments and devices whose validity and reliability have been examined and confirmed.
- **Reliability of Instruments/Data Collection:**
  All tests will be conducted using instruments and devices whose validity and reliability have been examined and confirmed.

**Sample Size and Calculation Method**

The sample size was calculated using G*Power software version 3.1. The calculations, based on a two-tailed test comparing the means of two dependent groups, with a type I error of 5%, a test power of 80%, and an effect size of 0.75, indicated that a total of 16 samples would be required. Given that the number of eligible patients referred to the clinic over the course of one year is approximately equal to this number, it is estimated that this sample size can be achieved within one year.

**Data Analysis Method**

Descriptive statistics, including mean and standard deviation or frequency and percentage, will be used to summarize the data. Comparisons before and after the intervention will be performed using appropriate parametric and non-parametric statistical tests, utilizing SPSS software version 26, with a significance level set at 5%.

**Ethical Considerations**

All ethical principles outlined in the Declaration of Helsinki will be observed in this study. Participation will be voluntary and conditional upon signing an informed consent form. Data and participant identities will be kept confidential. Any participant wishing to withdraw from the study at any stage may do so freely and without any coercion. The eye drops used in this study are considered safe and effective, with no significant adverse events reported in previous studies; however, if any complications occur, the research team will take the best possible therapeutic actions promptly. Additionally, all umbilical cord blood donor samples will be screened for transmissible viral pathogens such as HIV, syphilis, and hepatitis B and C in accordance with laboratory protocols. Participants will not incur any costs for ophthalmic examinations or related tests.

**Security and Confidentiality Measures**

The principal investigator and research team are committed to accurate execution, collection, classification, and preservation of all study data and results. The rights of Baqiyatallah University of Medical Sciences will be protected in all publications or protocols resulting from this study.

**Challenges and Limitations**

If any participant fails to fully cooperate, they will be excluded from the study and replaced by a suitable alternative.

**Registration of the Project Title in the IRCT (If Needed)**

Following acquisition of the ethics approval code, registration for a clinical trial code will be initiated on the IRCT website.

**Study Implementation Method (Execution Procedure and Data Collection)**

Following approval of the study by the Medical School and the Ethics Committee of Baqiyatallah University of Medical Sciences, eligible individuals presenting to Baqiyatallah Hospital who meet the inclusion criteria will be enrolled. Patients will be diagnosed with Sjögren’s syndrome by a rheumatology specialist, based on established rheumatologic criteria and clinical findings, including a combination of sicca symptoms and autoimmune features: T-cell activity (confirmed by positive salivary gland biopsy) or B-cell activity (presence of autoantibodies). These patients will then be referred to the ophthalmology clinic and, upon providing informed consent, will be enrolled in the study. Additionally, patients with moderate to severe chemical ocular injuries presenting with dry eye symptoms, as diagnosed by an experienced ophthalmologist, will be included.

For the control group, conventional treatments for dry eye, such as artificial tears and topical corticosteroid drops, will be administered and monitored for changes. Convenience sampling will be used. The purpose and procedures of the study will be clearly explained to all participants, and informed consent will be obtained.

Patients with Sjögren’s syndrome and chemical ocular injuries who have severe dry eye and have not responded adequately to conventional treatments—characterized by tear break-up time (TBUT) of less than 5 seconds, Schirmer’s test result of less than 5 mm, and positive corneal and conjunctival fluorescein staining (≥3)—will be included in the study.

After childbirth, umbilical cord blood will be collected immediately from consenting healthy mothers into a sterile, anticoagulant-free container. Prior to preparation, all cord blood donors must provide written informed consent. In the laboratory, cord blood samples will be screened for transmissible viral pathogens, including human immunodeficiency virus (HIV), syphilis, and hepatitis B and C.

Virus-negative umbilical cord blood is incubated at room temperature for 2–4 hours, followed by centrifugation. The yellow supernatant (serum) is carefully separated into a new sterile tube, and the red blood cell pellet and cellular debris are discarded. Heat inactivation of the collected serum is performed at 56°C for 30 minutes, followed by filtration and storage at –20°C for future clinical use.

Opened vials of serum eye drops are stored at 4°C in the refrigerator, and unopened vials are kept at –20°C. Each opened vial is used for one week. Serum eye drops are administered 4–6 times daily, and preservative-free artificial tears may be recommended as needed.

Assessment of dry eye symptoms, visual acuity, tear break-up time (TBUT), Schirmer’s test, and corneal and conjunctival fluorescein staining will be performed and documented at baseline, and at one and two months after treatment.

Subjective assessment of dry eye symptoms will be conducted using the validated Persian version of the Ocular Surface Disease Index (OSDI) questionnaire, which has been shown to be reliable and valid in the Iranian population (25).

Uncorrected and best-corrected visual acuity will be recorded at each follow-up using the logarithm of the minimum angle of resolution (logMAR) scale (26).

Schirmer’s test will be conducted using a standardized kit containing a 5 × 30 mm filter paper strip placed at the temporal edge of the lower eyelid. The patient is asked to look upward and blink normally for 5 minutes before the length of the wetted paper is measured. A wetting length of less than 5 mm is considered abnormal (27).

Tear film stability will be assessed using a slit lamp with cobalt blue filter and sodium fluorescein. One drop of 2% fluorescein is instilled into the eye, and the patient is instructed to blink five times to distribute the dye evenly. The patient is then asked to keep their eyes open without blinking, during which dry spots or black lines may appear, indicating tear film instability. The interval between the last blink and the appearance of the first dry spot is recorded as the tear break-up time (TBUT). The mean of three measurements is recorded. A TBUT of less than 10 seconds is considered abnormal (27).

According to the SICCA (Sjogren's International Collaborative Clinical Alliance) criteria, each eye’s cornea and both nasal and temporal conjunctiva are evaluated as separate regions. Corneal staining is scored from 0–6 and conjunctival staining from 0–3 for each region, resulting in a total conjunctival score of 0–6 and a combined ocular surface score of 0–12 per eye.

- **Corneal Staining:**
  - Score 0: No staining
  - Score 1: 1–5 dots
  - Score 2: 6–30 dots
  - Score 3: More than 30 dots
  - Additional points are given for continuous staining, central corneal staining, or filamentous staining, resulting in a maximum corneal score of 6.
- **Conjunctival Staining:**
  - For both nasal and temporal areas:
    - Score 0: 0–9 dots
    - Score 1: 10–32 dots
    - Score 2: 33–100 dots
    - Score 3: More than 100 dots
  - Total conjunctival score per eye ranges from 0 to 6 (28).

**Variables Table**

| No. | Variable | Role (Independent, Dependent, Background, Confounder) | Continuous | Discrete | Nominal | Ordinal | Scientific Definition | Operational Definition | Unit |
| --- | --- | --- | --- | --- | --- | --- | --- | --- | --- |
| 1 | Umbilical cord blood serum eye drop | Independent |  | * |  |  | Eye drops derived from healthy human umbilical cord blood, containing large amounts of cytokines, vitamin A, growth factors, neurotrophic factors, essential tear components, as well as antimicrobial and antifungal agents such as IgG. The drops are preservative-free, safe, and effective for tissue regeneration and repair. | Application (before/after intervention) | cc – before/after |
| 2 | Subjective symptoms | Dependent |  | * |  |  | Disease symptoms as reported by the patient. | Reported by the patient (e.g., OSDI questionnaire) | 0–100 |
| 3 | Visual acuity | Dependent | * |  |  |  | Defined as the eye’s ability to distinguish two objects as separate. Represents the minimal resolvable distance and the ability to identify gaps between two objects; assessed using standard visual acuity charts such as Snellen. | LogMAR value on standardized charts (e.g., Snellen chart) | logMAR |
| 4 | Tear break-up time (TBUT) | Dependent | * |  |  |  | The time required for the regular tear film layer on the cornea to break up. | Seconds measured from the last blink to the appearance of the first dry spot | Seconds |
| 5 | Schirmer’s test | Dependent | * |  |  |  | Schirmer’s test performed using a standardized filter paper strip (5 × 30 mm), placed on the temporal edge of the lower eyelid; measures tear production. | Millimeters of wetting over 5 minutes | mm |

**Implementation Steps and Timeline Table**

| No. | Implementation Step | Timeline by Month | Progress (%) | Phase Cost (%) |
| --- | --- | --- | --- | --- |
| 1 | Proposal approval and project initiation | * | 5.0 | 5.0 |
| 2 | Data collection | * * * * * * * * * * * * * * | 70.0 | 70.0 |
| 3 | Data analysis and dissemination of results | * * * * * | 25.0 | 25.0 |
