# Supplementary material for: Evaluation of umbilical cord blood serum eye drops for severe dry eye in two distinct populations—Sjögren’s syndrome and mustard gas–induced ocular injury: Protocol for a pilot randomized clinical trial
Source: PLoS One. 2025 Dec 4;20(12):e0337186. doi: 10.1371/journal.pone.0337186 (PMC12677545; doi:10.1371/journal.pone.0337186)
Supplement: S4 File — (DOCX) [file pone.0337186.s004.docx]

*Table 1- Project assessment and time steps.*

| **Procedures** | Screening  Day -1 | Intervention  Day -1 | Study Visit 1  Day 30 | Study Visit 2  Day 60 |
| --- | --- | --- | --- | --- |
| Written informed consent | X |  |  |  |
| Demographics | X |  |  |  |
| Medical history | X |  |  |  |
| Randomization | X |  |  |  |
| Administer study intervention |  | X |  |  |
| Concomitant medication review | X | X | X | X |
| Performance status | X |  |  |  |
| REFRACTION (OU) | X |  | X | X |
| BCVA (Log MAR) | X |  | X | X |
| OSDI questionnaire | X |  | X | X |
| TBUT (s) | X |  | X | X |
| Tear meniscus height (TMH) | X |  | X | X |
| SM tube test | X |  | X | X |
| Corneal staining (SICCA): | X |  | X | X |
| Conjunctiva staining TEMPO (SICCA) | X |  | X | X |
| Conjunctiva staining NASAL (SICCA) | X |  | X | X |
| Adverse event review and evaluation | X | X | X | X |
| Complete Case Report Forms (CRFs) | X | X | X | X |
